# Oxaliplatin, irinotecan and capecitabine as first-line therapy in metastatic colorectal cancer (mCRC): a dose-finding study and pharmacogenomic analysis

**DOI:** 10.1038/sj.bjc.6605595

**Published:** 2010-03-09

**Authors:** R Zarate, J Rodríguez, E Bandres, A Patiño-Garcia, M Ponz-Sarvise, A Viudez, N Ramirez, N Bitarte, A Chopitea, J Gacía-Foncillas

**Affiliations:** 1 Laboratory of Pharmacogenomics, Division of Oncology, CIMA, Pamplona, Spain; 2 Unit for the Research and Treatment of Gastrointestinal Malignancies, Department of Oncology, Pamplona, Spain; 3 Laboratory of Pediatrics, Department of Pediatrics, University Clinic of Navarra, Pamplona, Spain

**Keywords:** capecitabine, irinotecan, oxaliplatin, metastatic colorectal cancer, GSTP1

## Abstract

**Background:**

A dose-finding study was performed to evaluate the dose-limiting toxicity (DLT), maximum-tolerated dose (MTD) and the recommended dose (RD) of escalating the doses of capecitabine and fixed doses of irinotecan and oxaliplatin on a biweekly schedule for metastatic colorectal cancer patients (mCRC). A pharmacogenomic analysis was performed to investigate the association between SNPs and treatment outcome.

**Methods:**

Eighty-seven chemotherapy-naïve mCRC patients were recruited through a two-step study design; 27 were included in the dose-finding study and 60 in the pharmacogenomic analysis. Oxaliplatin (85 mg m^-2^) and CPT-11 (150 mg m^-2^), both on day 1, and capecitabine doses ranging from 850 to 1500 mg m^-2^ bid on days 1–7 were explored. Peripheral blood samples were used to genotype 13 SNPs in 10 genes related to drug metabolism or efficacy. Univariate and multivariate Cox analysis was performed to examine associations between SNPs, ORR and PFS.

**Results:**

The capecitabine RD was 1000 mg m^−2^ bid. Diarrhoea and neutropenia were the DLTs. After a median follow-up of 52.5 months, the median PFS and OS were 12 (95% CI; 10.6–13.4) and 27 months (95% CI; 17.2–36.8), respectively.

The GSTP1-*G* genotype, the Köhne low-risk category and use of a consolidation approach strongly correlated with decreased risk of progression. Patients with all favourable variables showed a median PFS of 42 months *vs* 3.4 months in the group with all adverse factors. A superior clinical response was obtained in patients with one GSTP1-G allele as compared with GSTP1-*AA* carriers (*P*=0.004).

**Conclusion:**

First-line therapy with oxaliplatin, irinotecan and capecitabine is efficient and well-tolerated. The GSTP1 polymorphism A>G status was significantly associated with ORR and PFS in mCRC treated with this triplet therapy.

Colorectal cancer (CRC) is one of the most common malignancies and the second leading cause of death from cancer in Europe and North America. It is responsible for approximately 1 million new cases and half a million deaths per year, worldwide (Bray *et al*, 2002).

Relevant advances have been made in the last years in the treatment of this disease, essentially owing to the introduction of combination chemotherapy regimens containing oxaliplatin (L-OHP) and irinotecan (CPT-11) (Kelly and Goldberg, 2005). Addition of either drug to 5-fluorouracil plus leucovorin (5-FU/LV) significantly increased both objective tumour response rate (ORR) and prolonged overall survival (OS) (de Gramont *et al*, 2000; Saltz *et al*, 2000). More recently, a triplet combination of 5-FU, L-OHP and CPT-11 has emerged as a feasible and highly active regimen for mCRC patients. We (Calvo *et al*, 2002a, 2002b) and others (Comella *et al*, 2002; Falcone *et al*, 2002) had previously reported a consistently high response rate and prolonged survival times with varying doses and schedules of this triplet regimen.

Pooled results of large-scale, phase-III trials have shown that first-line treatment of mCRC with capecitabine, an oral fluoropyrimidine pro-drug (Miwa *et al*, 1998), provided advantages over intravenous 5-FU/LV in terms of response rate and safety profile, with equivalent survival times (Hoff *et al*, 2001; Van Cutsem *et al*, 2001). Subsequently, several phase I and phase II trials tested capecitabine as the combination partner for L-OHP (Cassidy *et al*, 2004), CPT-11 (Tewes *et al*, 2003) or both (Souglakos *et al*, 2003), with preliminary promising results in terms of efficacy and a predictable toxicity profile.

On the basis of this rationale, we initiated a study with a dual aim: first to determine the optimal dose of capecitabine in combination with L-OHP and CPT-11 as first-line therapy for mCRC patients. The second objective was to identify potential polymorphisms related to a better and safer outcome.

In this study we report, after a significant long-term follow-up, the effect of both clinical and molecular parameters on the outcome of chemotherapy-naïve mCRC patients treated with this triplet regimen. We propose a combined clinical and pharmacogenomic assessment to identify which patients could benefit from this efficient regimen.

## Materials and methods

We initially performed a dose-finding study of escalating doses of capecitabine with biweekly fixed doses of CPT-11 and L-OHP in patients with mCRC to determine the dose-limiting toxicity (DLT), maximum-tolerated dose (MTD) and recommended dose (RD). Additional research was conducted to determine the activity and toxicity profile of the RD and to investigate the association between genetic polymorphisms and treatment outcome in terms of response rate and progression-free survival (PFS).

### Eligibility

All eligible patients had histologically confirmed mCRC not amenable to curative surgery, age between 18 and 75 years, an Eastern Cooperative Oncology Group (ECOG) performance status score of 0–2 and a life expectancy >12 weeks. In addition, adequate organ function was required as indicated by an absolute neutrophil count >1.5 × 10^9^/l, a platelet count >75 × 10^9^/l, serum creatinine <1.3 mg dl, serum bilirubin <1.25 times the normal upper limit (UNL) and serum transaminases <3.0 times UNL. The exclusion criteria included active second malignancy (except for non-melanoma skin cancer or *in situ* cervical cancer), symptomatic metastases in the central nervous system or carcinomatous leptomeningitis, uncontrolled severe infection, major organic failure, ischemic cardiopathy, active inflammatory bowel disease or chronic diarrhoea syndrome, bowel obstruction or prior extensive pelvic radiotherapy.

Pretreatment baseline evaluation included a complete medical history, physical examination, full blood count, biochemistry, including carcinoembrionic antigen, and a CT scan of the chest, abdomen and pelvis. During treatment, a physical examination and blood cell counts were performed biweekly. Treatment was delayed until recovery in the case of neutrophil level <1500/mm^3^, platelet level <75 000/mm^3^ or diarrhoea or stomatitis grade >1 on the planned day of treatment. If treatment had to be delayed for longer than 2 weeks, or any drug discontinued permanently, patients were excluded from the study. In the case of grade-3 to grade-4 adverse events, treatment was continued after resolution of the event using doses of L-OHP, CPT-11 and capecitabine reduced by 25%, except in the case of grade 3/4 diarrhoea, when only CPT-11 and capecitabine doses were reduced by 25%. The L-OHP dose was reduced by 25 or 50% in patients with grade-2 or grade-3 peripheral neuropathy, respectively. The capecitabine dose was reduced by 25% in case of grade 3/4 stomatitis and/or **H**and-**F**oot **S**yndrome.

Patients’ characteristics and their outcomes were unknown to investigators performing genetic analyses. The local institutional review board approved the study and all patients provided written informed consent before recruitment.

### Study design and treatment

Between April 2004 and October 2008, a total of 87 mCRC patients were enrolled in a two-step study.

### Step-1. Dose-finding study

Treatment consisted of escalating doses of capecitabine starting from the evening of day 1 to day 7, in association with fixed biweekly doses of L-OHP (85 mg m^−2^ infused over 2 h) immediately followed by CPT-11 (150 mg m^−2^ infused over 90 min). The L-OHP and CPT-11 doses and schedule were based on *in vitro* synergistic activity and on phase-I clinical trials combining the two drugs every two weeks, (Goldwasser *et al*, 2000), with a planned initial CPT-11 dose reduction due to the expected overlapping gastrointestinal toxicity with the addition of capecitabine. The planned dose levels of capecitabine were 850, 1000, 1250, and 1500 mg m^-2^, twice a day (Supplementary Table 1). Consecutive cohorts of at least three patients were included sequentially in each dose level, and no intra-patient dose escalation was allowed. If one out of three patients experienced a DLT, a minimum of three additional patients was enrolled at the same dose level. MTD was defined if two out of three or four out of six patients experienced a DLT. The recommended dose was the dose level just below the MTD.

All toxicities experienced during the study were recorded and graded according to the National Cancer Institute–Common Toxicity Criteria (NCI–CTC). DLT was defined as grade-4 neutropenia lasting more than 7 days, grade 3/4 neutropenia associated with fever >38°C, grade-4 thrombocytopenia, symptomatic thrombocytopenia (haemorrhage), and grade 3/4 non-haematological toxicity, except for alopecia, nausea or vomiting.

### Step-2. Clinical and pharmacogenomic analysis

Once the capecitabine RD was established in a limited dose-finding assessment, this cohort was further expanded to prospectively perform an exploratory pharmacogenomic profiling of the triplet combination. The sites of metastatic disease were radiologically re-evaluated every 8 weeks according to standard RECIST criteria unless clinically otherwise indicated (Therasse *et al*, 2000). At the time of maximum response, determined by serial CT scans or Positron Emission Tomography (PET) if clinically indicated, patients were evaluated by a multidisciplinary team that included surgeons, medical oncologists, hepatologists and interventional radiologists. In this evaluation it was ruled out whether a consolidative approach (CA) should be attempted. These approaches consisted of surgical removal of all macroscopic remaining disease, radiofrequency ablation, liver radioembolisation using Yttrium^90^ microspheres or cytoreductive surgery with hypertermic intraperitoneal chemotherapy (HIPEC).

### Genotyping

Genomic DNA was extracted from 200 *μ*l of whole blood using the DNA Isolation Kit-I from MagNa Pure LC (Roche, Barcelona, Spain) according to the protocols provided by the manufacturer.

Thirteen polymorphisms in 10 genes related to drug metabolism, involved in DNA repair or target of chemotherapy agents, were selected from various reports as being potentially predictive of oxaliplatin, fluoropyrimidines or irinotecan efficacy, and/or toxicity. The primer sequences and analysis technique are listed in Supplementary Table 3.

Some polymorphic regions were analysed by PCR-RFLP (restriction fragment length polymorphism) technique. Digestions were performed in a final volume of 50 *μ*l, containing 20 *μ*l of PCR product. Appropriate units of the corresponding enzymes were added to the other components provided by New England Biolabs (NEB, Beverly, MA, USA). After restriction enzyme digestion, fragments were visualised on a 2–3% agarose or LM-Sieve gel (Pronadisa, Madrid, Spain). Furthermore, one sample for each of the genotypes obtained by the RFLP technique was confirmed by direct sequencing the polymorphic region of each gene. No differences were obtained between both methods. The presence of the GSTT1 and GSTM1 genes was tested simultaneously using allele-specific sequence primers through a multiplex PCR protocol, according to the method previously described (Kim *et al*, 2000), with slight modifications. The albumin gene was used as positive control in each reaction. Analysis of the A(TA)nTAA motif in the promoter region of the UGT1A1 gene was performed by PCR, according to previous protocol (Monaghan *et al*, 1999), followed by automated sequencing analysis of the purified PCR products using an automated sequencer (ABI PRISM 3130XL DNA Sequencer; Applied Biosystems, Madrid, Spain).

The ERCC1-118 genotypes were determined using a TaqMan Allelic Discrimination Assay (Applied Biosystems, Foster City, CA, USA; C_8901525_10) according to the manufacturer's instructions. Briefly, 10 ng of DNA were added to 25 *μ*l of reaction containing forward and reverse primers along with two allele-specific labelled probes.

The number of 28-bp tandem repeats (2R or 3R) in the TYMS gene and the associated G>C SNP was performed as previously described (Kawakami and Watanabe, 2003; Mandola *et al*, 2003; Lincz *et al*, 2007). The polymorphism (G>C) in 5′-UTR region changes a crucial residue in the upstream stimulatory factor (USF) E-box consensus elements, abolish this USF binding and alter transcriptional activity. The classification of TYMS allele was based on the number of functional USF, consensus elements, in this 5 polymorphic region (Lincz *et al*, 2007). In our study, the most frequent allele was two and three USF sites (63 and 30%, respectively), whereas one or four USF sites were founded in only 2.7 and 4% of the patients, respectively. The favourable TYMS-5′-UTR genotype included those genotypes that have been linked to decreased TYMS expression (2C/2C, 2C/3C, 2GC/3C, 2C/3G, 3C/3C). On the contrary, unfavourable groups included three repeat genotypes with a G allele (2GC/3G, 3G/3C, 3G/3G). These genotypes were considered as a single class when statistical analysis was performed.

### Statistical analysis

A descriptive statistical methodology was used to design and analyse the dose escalation study. An exploratory pharmacogenomic analysis was conducted as an extension of the recommended dose level, where the primary endpoint was to correlate genetic polymorphisms with ORR and PFS. Secondary endpoints were aimed at finding out associations between SNPs, toxicity profile and OS. To calculate the sample size of the pharmacogenomic study, the expected frequency of unfavourable genotypes and its effect on RR and DFS was assessed. A Simon two-stage phase-II clinical trial design enrolling a minimum of 35 (stage-1) and a maximum of 60 eligible patients (stage-2) was chosen to get at least 25% of GSTP1-AA patients with a difference in terms of PFS greater that 1.5-fold with respect to genotypes harbouring any G-allele. With the significance level set at 0.05, the power to detect these differences was 80%.

Progression-free survival and OS were calculated from the first day of treatment to the date of first observation of progressive disease, death or last contact. Patients without progression at the time of the analysis were censored at their last available follow-up assessment. The log-rank test and Kaplan–Meier plots were used to evaluate the impact of genotypes on PFS.

Contingency tables and *χ*^2^-tests were used to check the association of response or toxicity with the polymorphic genes analysed. All *P-*values were two-sided. Compare2 from the WINPEPI program, version 1.42 (Copyright J.H. Abramson, 2003-5, part of the PEPI suite of computer **P**rograms for **EPI**demiologists), was used when the *χ*^2^-tests did not fulfil the analysis condition based to Cochran rule: If a *χ*^2^-test frequencies expected are 1 or less in more than 20% of the square, exact test 2 × K tables of PEPI program can be used.

The hazard ratio (HR) of clinical response and/or toxicity of the most relevant polymorphisms and patient outcome were assessed using Cox proportional hazard models using the reverse method. The model was adjusted taking into consideration relevant clinical parameters according to the univariate analysis and use of any consolidation approach that may likely influence on survival times (Gray *et al*, 2001; Verwaal *et al*, 2003, 2008; Adam *et al*, 2004; Poston *et al*, 2005). Confidence intervals (CIs) were within 95%.

All statistical tests were conducted using the SPSS software v15.0 for Windows (SPSS Inc., Chicago, IL, USA). Probability values (*P*-values) lower than 0.05 were considered statistically significant.

## Results

### Dose finding study

The number of patients in each dose level and the type of DLTs are summarised in Supplementary Tables 1 and 2. The starting dose level was L-OHP 85 mg m^−2^, CPT-11 150 mg m^−2^ and capecitabine 850 mg m^−2^, twice a day. At this initial dose level, grade-4 neutropenia lasting >7 days was recorded in one patient; so six more patients were included in this cohort. Only one out of these six patients presented with DLT (grade-4 diarrhoea and grade-3 febrile neutropenia). Three patients entered into the second dose level, and no DLT was observed. Dose escalation proceeded to dose level-3 (capecitabine 1250 mg m^−2^, twice a day). Two of three patients treated at this level developed DLT: grade-4 diarrhoea and grade-3 febrile neutropenia (one patient), and grade-4 diarrhoea with grade-3 asthenia (one patient). The RD was therefore established at dose level-2. This dose level was subsequently expanded, with only one of the 12 additional patients experiencing a DLT (grade-3 diarrhoea), (Supplementary Table 2).

### Clinical and pharmacogenomic analysis

In the second part of the study, 63 additional mCRC patients were subsequently treated with L-OHP 85 mg m^−2^, CPT-11 150 mg m^−2^ and capecitabine 1000 mg m^−2^, twice a day on days 1–7. Genotyping analysis was successful in 60 out of 63 patients. The clinicopathological characteristics of these patients are summarised in Table 1. Most of them had an ECOG 0–1, were classified as low- or intermediate-risk patients according to Köhne classification and had a low tumour burden (the median number of metastatic sites was 1). The median number of cycles administered was seven, with 26 patients receiving more than eight cycles.

The toxicity profile of the combination is listed in Table 2. The most common severe haematological toxicity was grade 3/4 neutropenia in 27% of the patients. Gastrointestinal disturbances were mild, although grade 3/4 diarrhoea and emesis were noted in 11 and 5% of the patients, respectively. Grade 1/2 neurosensory toxicity was developed in 67% of the patients, with only 2% reported as severe. Sixteen patients (26.7%) required a dose reduction and a dose delay was registered in 21 patients (35%).

On an intent-to-treat basis we observed a 66.6% ORR with 38 (63.3%) PR and 3 (3.3%) CR. Clinical response (CR, PR, and SD lasting >6 months) was achieved in 75% of the patients. Twenty-eight patients (46.7%) received a consolidation approach, including liver surgery (20 patients), liver radioembolisation with Yttrium^90^ microspheres (4 patients), liver radiofrequency ablation (1 patient) and peritonectomy with HIPEC (3 patients).

After a median follow-up of 52.5 months (range: 40.5–62.4), the median PFS and OS were 12 (95 % CI; 10.6–13.4) and 27 months (95 % CI; 17.2–36.8), respectively. Five-year PFS and OS were 10.4 and 23.9%, respectively.

#### Genetic determinants and response.

The observed allele frequencies were similar to those expected according to the Hardy–Weinberg equilibrium (data no shown).

Distribution of genotypes and response to chemotherapy are summarised in Table 3. For association analysis, responding patients were those with CR, PR, or SD lasting >6 months, whereas patients with SD less than 6 months were referred to as non-responders.

In univariate analysis, only GSTP1-variant (GG) was significantly related to response (*P*=0.007, χ^2^; Table 3, in bold). According to previous studies (Stoehlmacher *et al*, 2004) patients were classified as having a favourable genotype (homozygous variant GG and heterozygous AG) or an unfavourable genotype (homozygous AA). Patients harbouring at least one GSTP1-G allele showed significantly better clinical response rate as compared with patients with the GSTP1-AA genotype (79.5 *vs* 20.5%, *P*=0.004). None of the other genotypes analysed were significantly associated with treatment response. Among clinical parameters, only Köhne risk index (Köhne *et al*, 2002) was significantly associated with response (Table 1). Multivariate analysis showed GSTP1 as the only significant predictor of response (*P*=0.002).

#### Genetic determinants and toxicity.

For statistical analysis, overall toxicity was dichotomised as either moderate (grades 1–2) or severe (grades 3–4). Given the low incidence of severe adverse events we also tested genotype associations with grade 2/3 toxicity. Statistical analyses were performed by either considering each toxicity individually or by grouping them in three main categories: haematological, gastrointestinal and other.

No statistically significant differences with respect to toxicity were observed according to relevant clinical variables. Nevertheless, older patients (61–70 years) were more likely to develop grade-3 to grade-4 haematological and non-haematological toxicity (data no shown).

Considering toxicities on an individual basis, patients with the MTHFR c.1298 A/A genotype were more likely to develop grade 3/4 neutropenia (*P*=0.035). A significant association was also observed between the UGT1A1 6/7 genotype and grade 3/4 anaemia (*P*=*0.031*).

When grouped toxicities were considered, we found a statistically significant association between haematological toxicity and both UGT1A1-*6/7* and MTHFR c.1298 *A/A* genotypes (*P*=0.034; *P*=0.05, respectively). Haematological toxicity was also significantly related to UGT1A1 6/7 and 7/7 genotypes when toxicity grades were dichotomised between 0–1 and 2–4 (*P*=0.005) (data not shown). Development of gastrointestinal toxicity was also significantly related to UGT1A1-6/7 (*P*=0.001) and MTHFR c.1298-*AA* (*P*=0.023). A tendency to develop other non-haematological toxicities was also related to the MTHFR c.1298 *AA* genotype (*P*=0.079).

#### Genetic determinants and survival.

In the univariate analysis, of all clinical prognostic factors considered, only Köhne risk classification and consolidation approach were related to TTP and OS (Table 1).

**Figure 1:**
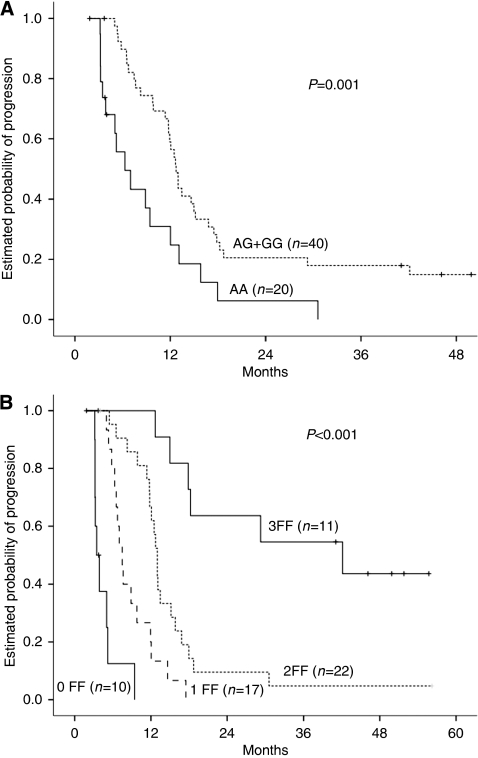
PFS Kaplan–Meier plot for PFS according to (**A**) the GSTP1-grouped genotype and (**B**) the number of favourable factors (FF): 2 years PFS for 0–1 FF, 0% and 5 years PFS for 2–3 FF, 5 and 42%, respectively.

A significantly higher risk of progression was associated with the GSTP1-AA genotype in the univariate analysis. Median PFS was 6.3 months for AA patients as compared with 12.7 months in the G-containing genotype patients (*P*=0.003) (Figure 1A; Table 4).

In the multivariate model, the GSTP1-G allele, the Köhne low-risk category and use of a consolidation approach were all associated with decreased risk of progression (Table 4) and were subsequently considered as favourable factors for further analyses. Patients with all favourable variables showed a median PFS of 42 months (95% CI; 5.8–78) *vs* 3.4 months (95% CI; 2.5–4.3) in the group with all adverse factors (GSTP1-AA, intermediate- or high-risk group according to Köhne classification and no consolidation approach) (*P*<0.001) (Figure 1B).

Finally, we sought to assess the influence of GSTP genotyping on the significant clinical variables in the multivariate model. Combined analysis of GSTP1-G allele with either consolidation therapy (Supplementary Table 4) or Köhne classification (Supplementary Table 5) was statistically significant when the different group of options were analysed.

Statistical differences were observed when OS was assessed according to the possibility of performing a consolidation approach (5-year OS; 39.5% *vs* 11.4%, *P*=0.001) or Köhne classification (5-year OS of 50.5% *vs* 0% for patients in the low- and high-risk category, respectively). In addition, 5-year OS was significantly longer for patients with the GSTP1-G genotype (25% *vs* 16%, *P*=0.02).

## Discussion

In this study, we have shown that the biweekly combination of L-OHP, CPT-11 and capecitabine is a feasible and active regimen, with neutropenia and diarrhoea as the DLT. The RD of capecitabine coincides with that recently reported by other authors with this same combination (Vasile *et al*, 2009). Neutropenia was frequently observed, although it was usually short-lived and rarely complicated. These findings are in contrast to those reported by Bajetta *et al* (2007) where no grade 3/4 neutropenia was reported. The different capecitabine dose (1000 mg/m^2^/day on days 2–6) might partly explain these discrepancies. Reported incidence of grade 3/4 diarrhoea in previous studies with this same triplet combination is 25–30%, (Masi *et al*, 2004; Bajetta *et al*, 2007) with CPT-11 doses of 165–180 mg m^−2^ every two weeks. The lower CPT-11 dose used in our study may be partly responsible of the two-fold decrease in the incidence of severe diarrhoea, without apparently compromising efficacy.

To the best of our knowledge, this is the first trial to explore the association between SNPs with a potential influence on L-OHP, CPT-11 and capecitabine and the clinical outcome with the use of a triplet cytotoxic regimen. So far, conflicting clinical findings exist regarding the predictive role of XPD-751, the combination of favourable genotypes within the TYMS-5′ and ERCC1 polymorphisms, and the efficacy of fluoropyrimidine/oxaliplatin-based chemotherapy (Stoehlmacher *et al*, 2004; Ruzzo *et al*, 2007; Martinez-Balibrea *et al*, 2008; Paré *et al*, 2008). In addition, a lack of association has been reported for the TYMS genotype and capecitabine-based therapy response (Tsuji *et al*, 2003; Martinez-Balibrea *et al*, 2008). However, our data suggest a role for the GSTP1 genotype as a predictive marker of clinical response and PFS. GSTP1-AA has been related with a higher enzymatic capacity for the conjugation of various cytotoxic drugs and a subsequent decrease of the cytotoxic effect of chemotherapy on tumour cells (Shen *et al*, 1997; O’Brien *et al*, 2000). Although initial results suggested longer survival for mCRC patients with the GSTP1-GG genotype who were treated with second- and third-line FOLFOX chemotherapy, (Stoehlmacher *et al*, 2002), more recent trials (Le Morvan *et al*, 2007; Ruzzo *et al*, 2007; Paré *et al*, 2008) found no association between GSTP1 variant and PFS. In addition, GSTP1 genotypes showed no clear influence on PFS in mCRC patients treated with either second-line based on XELOX or capecitabine as single agent (Kweekel *et al*, 2008, 2009). By contrast, a growing body of evidence suggests an association between GSTP1 and CPT-11 activity. *In vitro* experiments with human cell lines showed that when levels of nuclear GSTP1 are decreased, colonic HCT8 cells are partially more sensitive to CPT-11 (Goto *et al*, 2002). In the clinical setting (Kweekel *et al*, 2008), the survival benefit of adding CPT-11 to capecitabine for treatment of mCRC patients was only observed in those with the GSTP1-G-containing genotype. The results of this study seem to support these findings. Our patients harbouring at least one GSTP1-G allele had a reduced risk of progression of nearly three-fold and almost doubled the CRR as compared with patients with the GSTP1-AA genotype.

In an attempt to minimise possible confounding factors, we included in our analysis clinical parameters clearly related to patients’ outcome. It is noteworthy that the GSTP1 genotype had a significant influence on the PFS when these clinical parameters were also considered. Among patients who underwent liver surgery, a 5-year OS of 58% was obtained for GSTP1 AG/GG patients as compared with 0% for those patients who carried the AA genotype. Patients with the GSTP1-G-containing allele have also a prolonged median PFS within each of the Köhne risk categories. Combination of the GSTP1-G-containing genotype with the favourable clinical parameters identified patients who would benefit most from the use of this triplet scheme. In fact, a remarkable 5-year PFS of 43% was obtained for patients fulfilling all of these factors. In contrast, a median PFS of 3.4 and 7.4 months was obtained for patients with none or one favourable factor, respectively, suggesting that the up-front use of this triplet cytotoxic therapy may not be an optimal strategy in this setting. There may be several potential limitations in these findings. First, the limited sample size makes it mandatory for data to be confirmed in a larger cohort of patients. Second, although the Köhne risk index and the use of a consolidative approach seem to have a stronger effect on patients’ outcome as compared with the GSTP genotype, consolidative procedures cannot be used as a prospective marker for up-front determination of therapy benefit. Third, despite no differences between the frequencies of GSTP1 genotypes and patients undergoing liver surgery or a consolidative procedure were noted, patients carrying any G-containing allele were more likely to belong to the Köhne low- and intermediate-risk categories and therefore to receive any consolidative procedure as compared with those in the high-risk group, suggesting that these variables may not be truly independent.

According to other studies, grade 3/4 gastrointestinal toxicity was significantly more frequent in patients with the UGT1A1 6/7 genotypes (Liu *et al*, 2008). Although recent work suggests lack of association of UGT1A1^*^28 with irinotecan toxicity (Braun *et al*, 2009), the low incidence of the 7/7genotype (two patients) in our study precludes any firm conclusion. Our data also suggest that the MTHFR1298-AA is related to neutropenia and gastrointestinal toxicity. The link between this *MTHFR* polymorphism and toxicity remains controversial. Even if our results are not in agreement with previous reports (Zárate *et al*, 2007; Sharma *et al*, 2008), the reduced sample size or the low incidence of some grade 3/4 toxicities may have contributed.

The very significant long-term follow-up of our trial and the use of both clinical and molecular parameters may allow identification of patients who may benefit most from the up-front use of this triplet regimen. Confirmation of these findings, and particularly the very favourable outcome of patients with GSTP1-AG/GG, Köhne low-risk category and consolidation therapy with liver surgery, warrants further research.

## Figures and Tables

**Table 1:** Baseline characteristics of patients included in the pharmacogenomic analysis

		**Clinical response**			**TTP**		**OS**	
**Variables**	***n* (%)**	**Responder (CR+PR+SD>6 months)**	**Non-responder (SD <6 months)**	***P* a**	**Median (95% CI)**	***P* b**	**Median (95% CI)**	***P* b**
Age (years; range)	58 (37–75)	*n* (%)	*n* (%)					
<60	35 (56.5)	25 (71)	10 (29)	0.55	12 (8.1–15.8)	0.69	29 (14.5–43.6)	0.79
⩾60	27 (43.5)	20 (80)	5 (20)		12 (10.9–13.2)		24 (21–27)	
								
*Gender*								
Male	45 (75)	33 (73)	12 (27)	0.74	12 (10.9–13.2)	0.9	27 (17.6–36)	0.38
Female	15 (25)	12 (80)	3 (20)		9.8 (3.3–16.4)		24 (7.3–41.5)	
								
*ECOG performance status*								
0–1	54 (90)	39 (72)	15 (28)	0.26	12 (10.4–13.5)	0.79	27 (17.2–36.7)	0.31
2	6 (10)	6 (100)	—		12 (8.7–15)		15 (0.0–34.3)	
								
*Köhne risk classification*								
Low	26 (43)	22 (85)	4 (15)	0.04	15 (8.7–21.2)	0.001	35.7 (—)	0.001
Intermediate	28 (47)	21 (75)	7 (25)		11 (7.7–14.9)		24 (18.5–29.1)	
High	6 (10)	2 (33)	4 (67)		3 (1.3–5.1)		5 (3–6.8)	
								
*Primary tumour site*								
Colon	41 (68)	28 (68)	13 (32)	0.11	12 (9.2–14.6)	0.33	24 (21.3–27.4)	0.09
Rectum	19 (31)	17 (89)	2 (11)		13 (10.5–15.4)		43 (25.1–61.4)	
Previous radiotherapy	6 (10)	5 (83)	1 (17)	1	8 (5.3–9.8)	0.23	18 (16.1–29.6)	0.9
Previous adjuvant chemotherapy	12 (20)	10 (83)	2 (17)	0.71	9.8 (4.6–15.1)	0.31	19 (9.1–29.5)	0.9
								
*Consolidation*								
Yes	28 (48)	—	—	—	17 (12.2–21.4)	0.001	56 (32.9–79)	0.001
No	30 (52)	—	—		7 (5.8–7.6)		14 (5.1–22.7)	
Dose reduction	16 (27)	11 (69)	5 (31)	0.51	12 (4.8–19.2)	0.46	24 (12.7–35.8)	0.31
Dose delay	21 (35)	14 (67)	7 (33)	0.35	9.8 (2.2–17.4)	0.15	18 (4.8–31.9)	0.21

Abbreviations: CI=confidence interval; CR=complete response; ECOG=Eastern Cooperative Oncology Group; OS=overall survival; PR=partial remission; SD=stable disease.

a Clinical response calculated from *χ*^2^-test. Kaplan–Meier estimates of TTP and OS.

b Difference of the estimates tested using the log-rank test.

**Table 2:** Toxicity profile of the triplet combination

	**Grade**			
**Adverse event**	**1 (*n*, %)**	**2 (*n*, %)**	**3 (*n*, %)**	**4 (*n*, %)**
Leucopenia	15 (25)	20 (33)	2 (3)	3 (5)
Neutropenia	8 (13)	14 (23)	13 (22)	3 (5)
Anaemia	32 (53)	12 (20)	2 (3)	—
Thrombocytopenia	11 (18)	—	2 (3)	—
Vomiting	22 (37)	17 (28)	3 (5)	—
Diarrhoea	9 (15)	25 (42)	5 (8)	2 (3)
Mucositis	11 (18)	8 (13)	1 (2)	—
Asthenia	15 (25)	24 (40)	3 (5)	—
Neurotoxicity	25 (42)	15 (25)	1 (2)	—

**Table 3:** Distribution of genotypes according to response

		**Responder (CR+PR+SD> 6 months)**	**Non- responder (SD <6 months)**	
	**Total *n* (%)**	***n* (%)**	***n* (%)**	***P-*value**
All	60 (100)	45 (75)	15 (25)	—
				0.28
*TS-5-UTR*				
2R/2R	10 (16)	5 (50)	5 (50)	
2R/3R	31 (52)	23 (74)	8 (26)	
3R/3R	19 (32)	15 (79)	4 (21)	
				
*TS-5-UTR*+*G/C SNP*				0.056
A) 2C/2C,2C/3C, 2GC/3C,2C/3G,3C/3C	35 (61)	23 (66)	12 (34)	
B) 2GC/3G,3G/3C, 3G/3G	22 (39)	16 (73)	6 (27)	
				
*TS-31494del6*				0.26
−6/−6	6 (10)	5 (83)	1 (17)	
−6/+6	26 (43)	21 (81)	5 (19)	
+6/+6	28 (47)	19 (68)	9 (32)	
				
*GSTP1*				**0.007**
AA	20 (33)	10 (50)	10 (50)	
AG	34 (56)	30 (88)	4 (12)	
GG	6 (10)	5 (83)	1 (17)	
				
*GSTT1*				0.1
Present	46 (77)	34 (74)	12 (26)	
Null	14 (23)	11 (78)	3 (22)	
				
*GSTM1*				0.1
Present	25 (42)	21 (84)	4 (16)	
Null	35 (58)	24 (68)	11 (31)	
				
*GSTA1*				0.32
CC	20 (33)	15 (75)	4 (25)	
CT	28 (47)	21 (75)	7 (25)	
TT	13 (21)	9 (69)	4 (31)	
				
*XPD*				0.30
AA	23 (38)	18 (78)	5 (22)	
AC	30 (51)	21 (70)	9 (30)	
CC	7 (11)	6 (86)	1 (14)	
				
*XRCC1*				0.33
GG	27 (44)	20 (74)	7 (30)	
GA	26 (43)	20 (77)	6 (23)	
AA	7 (11)	5 (71)	2 (29)	
				
*ERCC1–118*				0.36
CC	9 (15)	6 (67)	3 (33)	
CT	25 (42)	21 (84)	4 (16)	
TT	25 (42)	17 (68)	8 (32)	
				
*UGT1A1*				0.38
5/6	1 (1.6)	—	1 (100)	
6/6	29 (48)	22 (76)	7 (24)	
6/7	28 (47)	21 (75)	7 (25)	
7/7	2 (3.3)	1 (50)	1 (50)	
				
*MTHFR-677*				0.17
CC	26 (43)	18 (69)	8 (31)	
CT	23 (38)	17 (74)	6 (26)	
TT	11 (18)	10 (91)	1 (9)	
				
*MTHFR-1298*				0.33
AA	31 (52)	22 (71)	9 (29)	
AC	23 (38)	18 (78)	5 (22)	
CC	6 (10)	5 (83)	1 (17)	

Abbreviations: CR=complete response; PR=partial remission; SD=stable disease; UTR=untranslated region.

**Table 4:** Adjusted Cox multivariate analysis for PFS

**Factor**	**Variable**	***n***	**PFS (median) months**	**Hazard ratio^a^**	**95% CI**	***P***
Köhne	Low	26	15	1.0	Reference	0.009
	Intermediate	28	9.8	2.5	1.2–5.1	
	High	6	3.2	15.9	4.3–59.1	
CA^b^	No	30	6.5	10.7	4.6–25.1	0.001
	Yes	28	16.8	1.0	Reference	
GSTP1	AA	20	6.3	2.6	1.3–5.4	0.008
	AG+GG	40	12.7	1.0	Reference	

Abbreviations: CI=confidence interval; GSTP1=glutathione-*S*-transferase Pi; HR=hazard ratio; PFS=progression-free survival.

a HR adjusted by significant genotypes (univariate analysis), Köhne classification.

b Consolidation approach after triplet treatment.
