# Supplementary material for: Oxaliplatin, irinotecan and capecitabine as first-line therapy in metastatic colorectal cancer (mCRC): a dose-finding study and pharmacogenomic analysis
Source: Br J Cancer. 2010 Mar 9;102(6):987–94. doi: 10.1038/sj.bjc.6605595 (PMC2844042; doi:10.1038/sj.bjc.6605595)
Supplement: Supplementary Tables 1–5 [file 6605595x1.doc]

**Supplementary table 1.** Capecitabine dose levels

| **Dose** (mg/ m^2^/ bid) | **N** | **Number of patients with** ^a^**DLT** | Type of DLT | **Efficacy** |
| --- | --- | --- | --- | --- |
| 850 | 9 | 2 | Grade 4 neutropenia  Grade 4 diarrhoea  Grade 3 febrile neutropenia | 7 ^b^PR, 2 ^c^SD |
| 1000 | 15 | 0 | - | 11 PR, 4 SD |
| 1250 | 3 | 2 | Grade 4 diarrhoea  Grade 3 febrile neutropenia  Grade 3 asthenia. | 1 PR, 2 SD |

^a^DLT; Dose limiting toxicity. ^b^PR; Partial response. ^c^SD; Stable disease.

## Supplementary table 2. Worst grade toxicity per patient

| **Event** | **Level 1 (n=9)** | **Level 2 (n=15)** | **Level 3 (n=3)** |
| --- | --- | --- | --- |
| **Leucopenia** |  |  |  |
| Grade 1-2 | 7 | 6 | 1 |
| Grade 3-4 | 1 | 0 | 1 |
| **Neutropenia** |  |  |  |
| Grade 1-2 | 4 | 5 | 0 |
| Grade 3-4 | 4 | 1 | 2 |
| **Anaemia** |  |  |  |
| Grade 1-2 | 7 | 9 | 3 |
| Grade 3-4 | 1 | 0 | 0 |
| **Thrombocytopenia** |  |  |  |
| Grade 1-2 | 5 | 0 | 3 |
| Grade 3-4 | 0 | 0 | 0 |
| **Diarrhea** |  |  |  |
| Grade 1-2 | 4 | 9 | 1 |
| Grade 3-4 | 1 | 1 | 2 |
| **Vomiting** |  |  |  |
| Grade 1-2 | 5 | 10 | 3 |
| Grade 3-4 | 0 | 0 | 0 |
| **Asthenia** |  |  |  |
| Grade 1-2 | 6 | 9 | 3 |
| Grade 3-4 | 1 | 0 | 1 |
| **Neurotoxicity** |  |  |  |
| Grade 1-2 | 5 | 11 | 3 |
| Grade 3-4 | 1 | 0 | 0 |
| **Fever** |  |  |  |
| Yes | 3 | 3 | 1 |
| No | 6 | 12 | 2 |

**Supplementary table 3.** Primer sequences, restriction enzymes and polymorphism characteristics

^a^ Restriction enzyme

| **Primers sequences (5´🡪3´)** | **RE**^a^ | **Variation** | **Significance** | **Reference** |
| --- | --- | --- | --- | --- |
| GSTA1 (rs3957357)  F🡪 AGAATCCAGTAGGTGGCCCC  R🡪 TGTTAAACGCTGTCACCGTCC | *EarI* | (C-69T)  (promotor region) |  | L13269^b^ |
| ***GSTP1*-105** *(rs1695)*  F🡪 ACCCCAGGGCTCTATGGGAA  R🡪 TGAGGGCACAAGAAGCCCCT | *BsmAI* | Ile🡪Val (A>G)  (Exon 5) | Low activity | (Harries *et al*, 1997) |
| GSTT1  F🡪 TTCCTTACTGGTCCTCACATCTC  R🡪 TCACCGGATCATGGCCAGCA | *-* | Gene deletion | Activity abolished | (Kim *et al*, 2000) |
| GSTM1  F🡪 CTGCCCTACTTGATTGATGGG  R🡪 CTGGATTGTAGCAGATCATGC | *-* | Gene deletion | Activity abolished | (Kim *et al*, 2000) |
| TYMS 5´ ^c^([**rs34743033**](http://www.ncbi.nlm.nih.gov/projects/SNP/snp_ref.cgi?rs=34743033))  F🡪 GTGGCTCCTGCGTTTCCCCC  R🡪 GGCTCCGAGCCGGCCACAGGCATGGCGCGG | *-* | 28 bp repeat  (5´-region) | Change of expression | (Horie *et al*, 1995) |
| TYMS 5´ ^c^ (rs2853542) | *HaeIII* | G>C  (into 28bp repeat 5´-region) | Affect USF*^d^* site | (Lincz *et al*, 2007) |
| TYMS-3´1494del6 ([**rs34489327**](http://www.ncbi.nlm.nih.gov/projects/SNP/snp_ref.cgi?rs=34489327))  F🡪 CAAATCTGAGGGAGCTGAGT  R🡪 CAGATAAGTGGCAGTACAGA | *DraI* | 6 bp deletion  (3´UTR) | Unknown | (Ulrich *et al*, 2000) |
| ***MTHFR*-677** *(rs1801133)*  F🡪 TGAAGGAGAAGGTGTCTGCGGGA  R🡪 AGGACGGTGCGGTGAGAGTG | *HinfI* | Ala222Val (C>T)  (exon 4) | Low activity | (Yi *et al*, 2002) |
| ***MTHFR*-1298** *(rs1801131)*  F🡪 CAAGGAGGAGCTGCTGAAGA  R🡪 CCACTCCAGCATCACTCACT | *MboII* | Glu433Ala (A>C)  (exon 7) | Low activity | (Yi *et al*, 2002) |
| ***XRCC1*** *(rs25487)*  F🡪 TTTGCCCCTCAGATCACACC  R🡪 TAAGGAGCAGGGTTGGCGT | *MspI* | Arg399Gln  G>A  (exon 10) | Low activity | (Lunn *et al*, 1999) |
| XPD (rs13181)  F🡪 ATCCTGTCCCTACTGGCCATT  R🡪 TGTGGACGTGACAGTGAGAAAT | *PstI* | Lys751Gln  A>C  (exon 23) | Low activity | (Dybdahl *et al*, 1999) |
| ***ERCC1*** ^e^ *(rs11615)* | *-* | Asn118Asn  C>T  (exon 4) | Low protein levels and activity | (Yu *et al*, 2000) |
| UGT1A1*28 (rs8175347)  F🡪 AAGTGAACTCCCTGCTACCTT  R🡪 CCACTGGGATCAACAGTATCT | Sequencing | A(TA)n TAA | Low glucuronidation | (Monaghan et al, 1999) |

^b^ GenBank sequence of the human *GSTA1*  gene. Primers were designed with the Primer Express 2.0 software.

^c^ Combined genotype analysis (21).

^d^ USF= operative binding sites of a transcription factor of TYMS

^e^ Pre-designed assays from Applied Biosystems.

**Supplementary table 4.** Combined analysis of unadjusted GSTP1 genotype and liver surgery as main consolidation therapy.

| **Liver surgery** | **n** | **GSTP1-grouped** | **PFS (median) months** | SE**^*^(95% CI)** | **p(LR test)** |
| --- | --- | --- | --- | --- | --- |
|  | 4 | AA | 13.1 | 6 (6.2; 19.9) | **0.003** |
| Yes (21) | 17 | AG+GG | 17.8 | 1.4 (15.1; 20.6) |
|  |  |  |  |  |
| **No** (38) | 15 | AA | 5.2 | 1.0 (3; 7.3) |
|  | 23 | AG+GG | 9.8 | 2.4 (5; 14.7) |

^*^SE= standard error.

**Supplementary table 5.** Combined analysis of unadjusted GSTP1 genotype and Köhne classification

| **Risk** | **n** | **GSTP1-grouped** | **PFS (median) months** | SE**^*^(95% CI)** | **p(LR test)** |
| --- | --- | --- | --- | --- | --- |
| Low (26) | 9 | AA | 13.1 | 1.3 (10.3; 15.8) |  |
|  | 17 | AG+GG | 17.8 | 3.8 (10.3; 25.3) |  |
| **Intermediate** (28) | 6 | AA | 3.8 | 0.9 (1.9; 5.7) | **0.001** |
|  | 22 | AG+GG | 11.9 | 1.0 (9.8; 13.9) |  |
| **High** (6) | 5 | AA | 3.2 | 0.03 (3.1; 3.3) |  |
|  | 1 | AG+GG | 9.8 |  |  |

^*^SE= standard error.
